# Clinical study of drug-loaded calcium sulfate in the treatment of hematogenous osteomyelitis in children

**DOI:** 10.1186/s12891-023-06948-z

**Published:** 2023-10-17

**Authors:** Dun Liu, Aierken Rehemutula, Yu Si, Hongyu Zhou, Jingyang Li, Zihao Chen, Li Li

**Affiliations:** Department of Orthopedics, Traditional Chinese Medical Hospital of Xinjiang Uygur Autonomous Region, The Fourth Affiliated Hospital of Xinjiang Medical University, Urumqi, Xinjiang China

**Keywords:** Hematogenous osteomyelitis, Children, Drug-laden calcium sulfate, Vancomycin

## Abstract

**Background:**

At present, good results have been obtained in the treatment of hematogenous osteomyelitis(HO) in children by the use of drug-loaded calcium sulfate, but there are few clinical studies reported. The aim of this study was to investigate the clinical efficacy of radical debridement combined with drug-laden calcium sulphate antibiotics in paediatric haematogenous osteomyelitis.

**Methods:**

In this study, we retrospectively analyzed the clinical data of 15 cases of pediatric hematogenous osteomyelitis admitted to our hospital in recent years. A total of 15 pediatric patients with HO treated in our hospital from January 2018 to February 2022 were included for evaluation.

**Results:**

All 15 patients were treated with drug-laden calcium sulfate, and the antibiotic of choice was vancomycin in 14 cases and vancomycin combined with gentamicin in 1 case. The follow-up period ranged from 12 to 36 months, with a mean follow-up time of 24.73 months, and all children were treated with drug-laden calcium sulfate with satisfactory clinical outcomes. The results of serological examination showed that the preoperative white blood cell count level, C-reactive protein and erythrocyte sedimentation rate were higher than the postoperative ones, and the differences were statistically significant (*P* < 0.05).After the operation, referring to the treatment standard of McKee’s osteomyelitis, 15 cases were cured without recurrence; According to the Lower Extremities Functional Scale, 12 cases were excellent, 2 cases were good and 1 case was moderate, with an excellent rate of 93.33%. Children with lower limb involvement could walk with full weight bearing, and gait was basically normal.

**Conclusion:**

Drug-loaded calcium sulfate is a good therapeutic method for the treatment of hematogenous osteomyelitis in children, with a effect of reducing complications and reducing recurrence.

## Background

Hematogenous osteomyelitis (HO) is a kind of osteomyelitis, occurs mostly in children compared with young adults, and its causative organisms are diverse but Staphylococcus aureus is the most common; Its susceptible site is the long bone epiphysis, which requires effective treatment and control by a physician after diagnosis, otherwise its infection tends to involve the whole bone, followed by the formation of dead bone, eventually causing bone defects and decreased or complete loss of joint (site) function [1–3]. The pathogenesis of HO is primarily an osteomyelitis caused by the spread of pathogenic bacteria through the bloodstream to reach the bone. The severity of HO is related to a number of factors, such as age, level of immunity, type of pathogen, site of disease, location of the lesion, and surrounding tissues. According to the course of disease, Acute haematogenous osteomyelitis is generally defined as an infection of less than 2 weeks, subacute as 2 weeks to 12 weeks (3 months) and chronic as 12 weeks [4]. If acute hematogenous osteomyelitis without effective intervention progresses to chronic hematogenous osteomyelitis after the formation of dead bone, it will continue to affect bone growth and development. Because chronic infections such as osteonecrosis often have poor blood flow around the lesion, patients often require multiple courses of long-term antibiotic therapy or surgical intervention [5]. Drug-laden calcium sulphate has been used in the treatment of HO in children, but the method and effectiveness of the treatment are not well defined, and there are few studies on the treatment of paediatric haematogenous osteomyelitis with drug-laden calcium sulphate at home and abroad [6, 7]. Therefore, as a complement, we treated paediatric haematogenous osteomyelitis by radical debridement combined with drug-loaded calcium sulphate, and further explored its clinical efficacy.

## Method

### Study design and setting

Retrospective analysis of children with HO were conducted at our hospital from January 2018 to February 2022. Eighteen cases were retrieved on the medical record system based on keywords, and 15 eligible patients were screened according to the inclusion and exclusion criteria, and the remaining 3 cases were lost to follow-up. The main inclusion criteria were 1) patients under 18 years of age with imaging findings (radiographs suggesting local soft tissue swelling, bone destruction or ultrasound showing subperiosteal fluid or magnetic resonance imaging suggesting bone marrow congestion, edema, oozing, etc.) Suggestive of HO; 2) patients who underwent surgical intervention and drug-laden calcium sulfate implantation; 3) bone defects formed after radical debridement; and 4) follow-up time ≥ 12 months. The main exclusion criteria were: 1) patients treated with immunosuppressive therapy or chemotherapy; 2) patients with cardiovascular and neurological diseases and psychological disorders affecting the prognosis of surgery; 3) patients with allergy to calcium sulfate; 4) patients who could not cooperate with the treatment or who were lost to follow-up. Clinical data collected included age, gender, clinical history, preoperative assessment data, serum inflammatory indexes (white blood cell count (WBC), erythrocyte sedimentation rate (ESR), C-reactive protein (CRP) level), bacterial culture results, etiology, imaging data, location of osteomyelitis (left/right), number of surgeries, surgery-related data, type of drug-laden calcium sulfate antibiotics, and follow-up time.The studies involving human participants were reviewed and approved by Hospital of Traditional Chinese Medicine affiliated to Xinjiang Medical University. Written informed consent to participate in this study was provided by the participants’ legal guardian/next of kin.

### Surgical methods

#### Preoperative

After admission, the relevant preoperative examinations were actively perfected, the location and extent of infection were confirmed by imaging examination (X-ray, CT or MRI), surgical contraindications were excluded by laboratory examination (blood routine, coagulation four items, biochemical panel, erythrocyte sedimentation rate, C-reactive protein, blood grouping, etc.), and bacterial culture and drug sensitivity test were performed on the secretions of the lesion area to determine the types of drug-loaded calcium sulfate antibiotics used, and the selection of postoperative anti-infective drugs. A surgical plan defining the extent of radical debridement was also developed.

#### During the operation

Intravenous inhalation combined with general anaesthesia and combined spinal-epidural anaesthesia was used. The patient was routinely positioned and the affected limb routinely was disinfected after the anaesthetic had taken effect. A sterile towel was draped, the tourniquet was used to control, the incision position was taken, the lesion was incised layer by layer to expose the lesion, the depth of the lacuna was explored by the probe, and some purulent secretions, surrounding tissues, and sequestrum scraped in the deep layer were taken for bacterial culture, drug sensitivity test, and pathological examination. Dead bone and sclerotic bone, inflammatory granulation tissue, and infected necrotic tissue were completely removed from the trauma. The trauma was flushed with plenty of saline, and the distal and proximal parts of the bone site and the upper and lower boundaries were located under C-arm fluoroscopy, and an external fixation frame can be used according to the site, size and needs of the bone defect. A carrier calcium sulfate mixed with antibiotics, properly shaped and implanted in the bone defect, washed again, drainage tubes may be left in place, absorbent thread and silk thread with stitches are sutured layer by layer to close the wound, and a sterile dressing was applied.

#### Postoperative

All 15 pediatric patients were treated with drug-loaded calcium sulfate, 14 selected antibiotics were vancomycin, and 1 was vancomycin combined with gentamicin. Intravenous antibiotics were routinely administered to all patients postoperatively. Bacterial cultures of all 15 patients were methicillin resistant Staphylococcus aureus (MRSA) infections. Five cases were treated with vancomycin 40 mg/kg/d against infection, four cases with cefazolin sodium and three cases with cefuroxime sodium 100 mg/kg/d. Two patients allergic to cefazolin sodium and cefuroxime sodium were treated with clindamycin 30 mg/kg/d and one case combined with linezolid combined with ceftriaxone sodium 100 mg/kg/d against infection. All patients received postoperative intravenous anti-infection for 7 days and oral antibiotics for 2 weeks (Table 1). Postoperatively, the status of the soft tissues was determined based on physical examination, and whether the infection had been eradicated was determined by radiographs and the results of c-reactive protein (CRP), erythrocyte sedimentation rate (ESR), and white blood cell count (WBC). The external fixation brace was used to allow the patient to be on the floor earlier and to achieve early mobility. The external fixation brace was removed on time according to x-ray showing fracture healing, and the patient was instructed to perform late rehabilitation functional training. The efficacy was evaluated postoperatively with reference to the McKee osteomyelitis treatment criteria [8] and the Lower Extremities Functional Scale (LEFS).

**Table 1:** Patient data

Patient number	Age	sex	Bone	Side	Disease time	Time of onset (months)	Symptoms	Drug calcium sulfate species	Causative agent	Anti-infection drugs
1	11	F	The talus bone, the calcaneus	R	In January, 2020	24	Red swelling, skin rupture, and sinus tract formation	vancomycin	MRSA	Cephazolin sodium
2	6	F	thigh-bone	L	In August, 2019	15	Red and swollen, painful	vancomycin	MRSA	Cephazolin sodium
3	14	M	thigh-bone	L	In July, 2019	24	dull pain	Vancomycin was combined with gentamicin	MRSA	clindamycin
4	7	M	calcaneus	L	In February, 2021	12	Red and swollen, painful	vancomycin	MRSA	Cephazolin sodium
5	5	M	tibiofibula	R	In July, 2020	38	Red, pain, skin rupture, sinus formation	vancomycin	MRSA	Cephazolin sodium
6	10	M	shin bone	R	In December, 2020	24	Pain, swelling	vancomycin	MRSA	Cefuroxime sodium
7	14	M	tibiofibula	R	In July, 2021	36	Pain, swelling	vancomycin	MRSA	Cefuroxime sodium
8	11	F	shin bone	L	In June, 2018	36	Red, pain, fever	vancomycin	MRSA	vancomycin
9	3	M	calcaneus	R	In April, 2020	12	Red, pain, skin rupture, sinus formation	vancomycin	MRSA	clindamycin
10	14	F	Metatarsal, phalanges, dice bones	R	In June, 2020	36	Red swelling, skin rupture, and sinus tract formation	vancomycin	MRSA	Cefuroxime sodium
11	15	F	shin bone	L	In December, 2017	24	Red, pain, skin rupture, sinus formation	vancomycin	MRSA	vancomycin
12	13	M	shin bone	R	In July, 2020	24	Red swelling, pain, swelling, and fever	vancomycin	MRSA	Linezolid combined with ceftriaxone sodium
13	15	M	thigh-bone	L	In August, 2019	36	Red swelling, pain, swelling, fever, and skin rupture	vancomycin	MRSA	vancomycin
14	17	F	Metatarsal, tibia	L	In June, 2020	24	Red swelling, pain, skin rupture, the formation of a sinus tract, fever	vancomycin	MRSA	vancomycin
15	12	F	shin bone	R	In December, 2021	12	Pain, swelling, and skin rupture	vancomycin	MRSA	vancomycin

*Abbreviation:* Methicillin-resistant Staphylococcus aureus (*MRSA*)

### Statistical analysis

Statistical analysis of the data obtained was performed using R4.1.0. Continuous variables were expressed as mean ± standard deviation (-x± *s*), and the preoperative and postoperative differences of different indicators conformed to the normal distribution, which was analyzed using the two-paired sample t-test, and those that did not conform to the normal distribution were analyzed using the two-paired sample Wilcoxon signed-rank test.

## Results

**Fig. 1:**
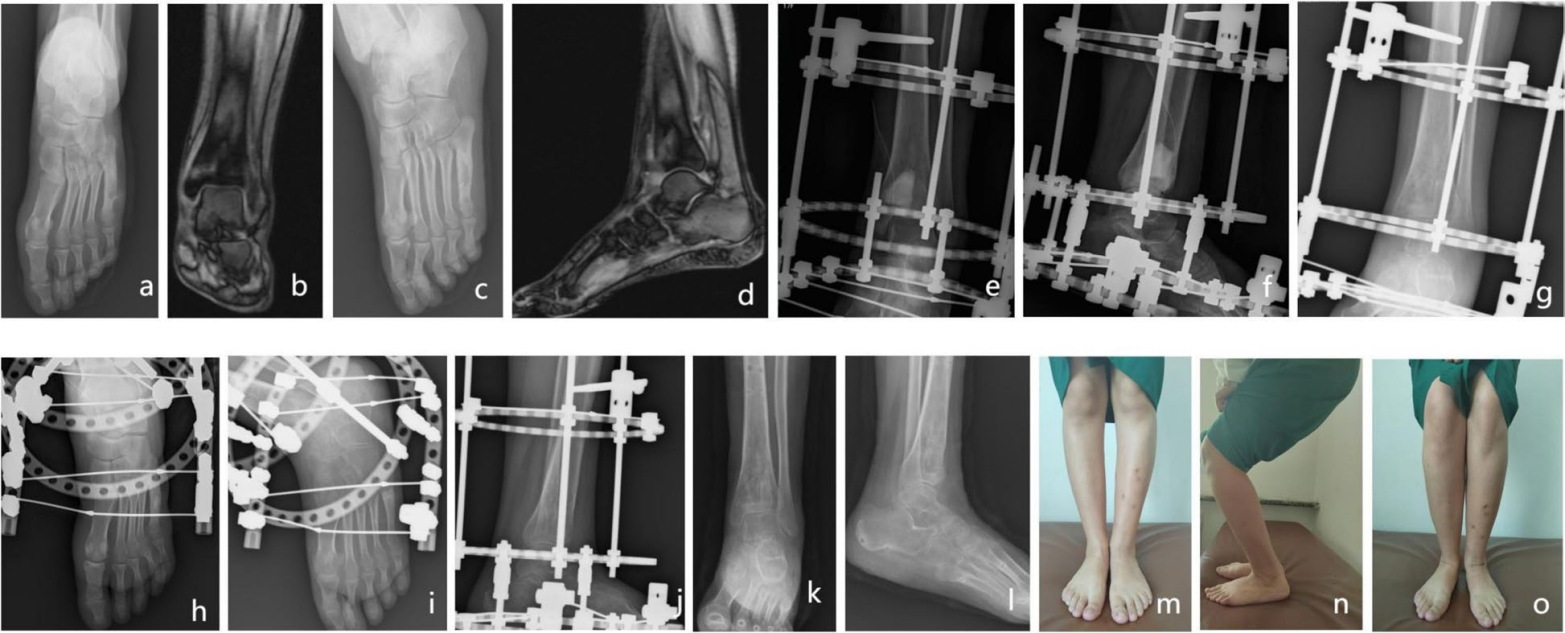
Patient, female, 17 years old, Osteomyelitis of the left metatarsal and left tibial bones with no apparent cause. **a**-**d** Preoperative X-ray and CT examination showed infection of the metatarsal and tibia; **e**–**h** Radical debridement, drug-loaded calcium sulfate implantation and external fixation showed anastomosis of the drug-loaded calcium sulfate with the bone defect site and stability of the external fixation frame on X-ray examination 1 day after surgery; **i**-**j** Good healing of the bone defect site on X-ray examination before removal of the external fixation frame 6 months after surgery; **k**-**l** Good surgical recovery on X-ray examination 7 months after surgery and 1 month after removal of the external fixation frame; **m**–**n** 24 months post-operative, full weight-bearing of the affected limb and almost normal gait

**Fig. 2:**
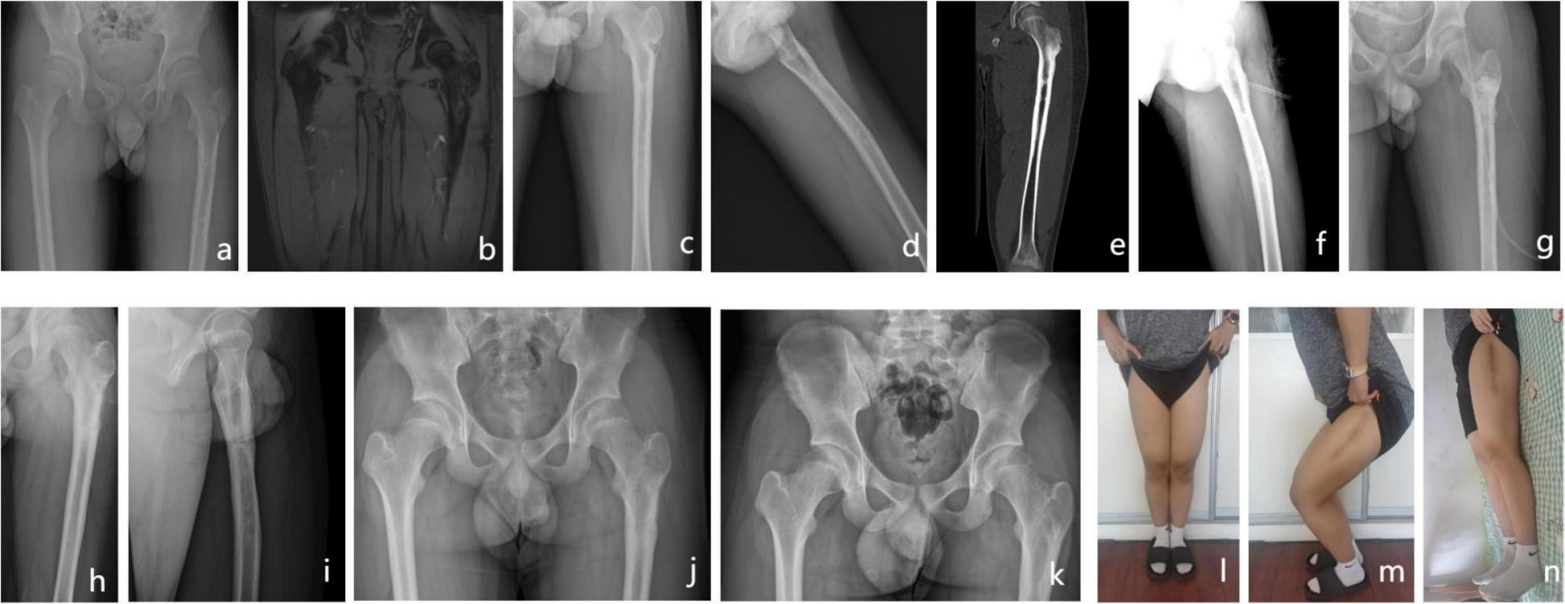
Patient, male, 15 years old, presented with osteomyelitis of the left femur with no obvious causative factors. **a**-**e** preoperative X-ray and CT examination showed infection of the left femur; **f**-**g**, **h**-**i** radical debridement and drug-laden calcium sulfate implantation showed anastomosis of the drug-laden calcium sulfate to the bone defect site on X-ray 1 and 3 days after surgery; **j** 6-month postoperative X-ray examination showed good healing of the bone defect site; **k**-**n** Post-operative X-ray at 36 months shows good post-operative recovery with full weight bearing of the affected limb and generally normal gait

Among the 15 children with HO, 8 were males and 7 were females, with a mean age of presentation of 11.13 (3 – 17) years, 8 (53.33%) were 3 to 12 years, and 7 (46.67%) were 13 to 18 years. Osteomyelitis was partially right in 8 patients (53.33%) and left in 7 patients (46.67%). It occurred in the tibia in 5 patients (33.3%), femur in 3 patients (20%), tibia and fibula in 2 patients (13.3%), calcaneus in 2 patients (13.3%), and occurred in multiple parts in 3 patients (20%), including talus and calcaneus in 1 patient, metatarsal, phalanges, and cuboid in 1 patient, and metatarsal and tibia in 1 patient. Most of the symptoms were redness, swelling, pain, skin ulceration and sinus formation, while a few were accompanied by fever, and only one case showed dull pain. The follow-up time ranged from 12 to 36 months, with an average of 24.73 months. After treatment with drug-loaded calcium sulfate, all patients obtained satisfactory clinical efficacy (Table 1). After the operation, referring to the treatment standard of McKee’s osteomyelitis, 15 cases were cured without recurrence; According to the Lower Extremities Functional Scale, 12 cases were excellent, 2 cases were good and 1 case was moderate, with an excellent rate of 93.33%. Children with lower limb involvement can walk with full weight bearing, gait is basically normal (Table 2).Typical case pictures are shown in (Figs. 1 and 2).

**Table 2:** Lower extremity function scale

Lower Extremities Functional Scale (LEFS)					
Scoring options and scores (n of patients = 15)	Extreme difficulty or inability to perform the activity score 0	Very difficult at 1 point	Medium difficulty of 2 points	A little difficult: 3 points	It’s not 4 points
a. Daily work, household activities	0	0	1	0	14
b. Regular entertainment or physical activity activities	0	1	1	0	13
c. Go into the bathroom by yourself	0	0	0	1	14
d. Walking in the room	0	0	0	0	15
e. Wear shoes or socks	0	0	0	0	15
f. squat on the heels	0	0	1	1	13
g. Lift the heavy object on the floor	0	0	0	2	13
h. Do mild activity at home	0	1	0	1	13
i. Do a high-intensity activity at home	1	2	1	1	10
j. Get on and off the bus	0	0	1	1	13
k. Get on and off the bus	0	0	0	0	15
l. Walk a kilometer	0	1	1	1	12
m. Up or down the floor	0	0	1	1	13
n. Stand for 1 h	0	1	3	1	10
o. Sit for an hour	0	0	1	1	13
p. Run in a flat place	0	1	1	2	11
q. Run in an uneven place	1	1	1	1	11
r. A sharp turn to run	1	1	1	1	11
s. jump	0	0	2	1	12
t. turn over	0	0	0	0	15

The score is 80,0–4 for each item, 0, very difficulty 1, medium difficulty 3, no difficulty 4 points, evaluation criteria: excellent 70–80; good 60–69; medium 50–59; difference < 50 points

**Fig. 3:**
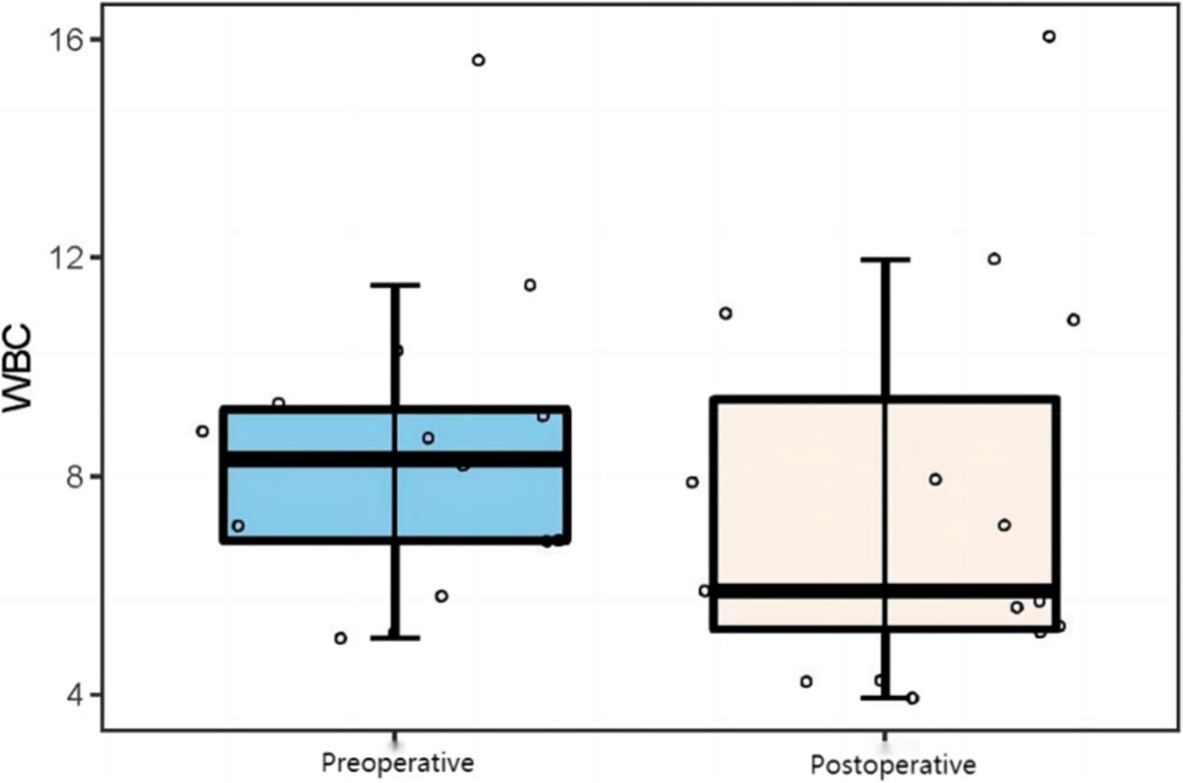
The WBC levels before and after surgery

**Fig. 4:**
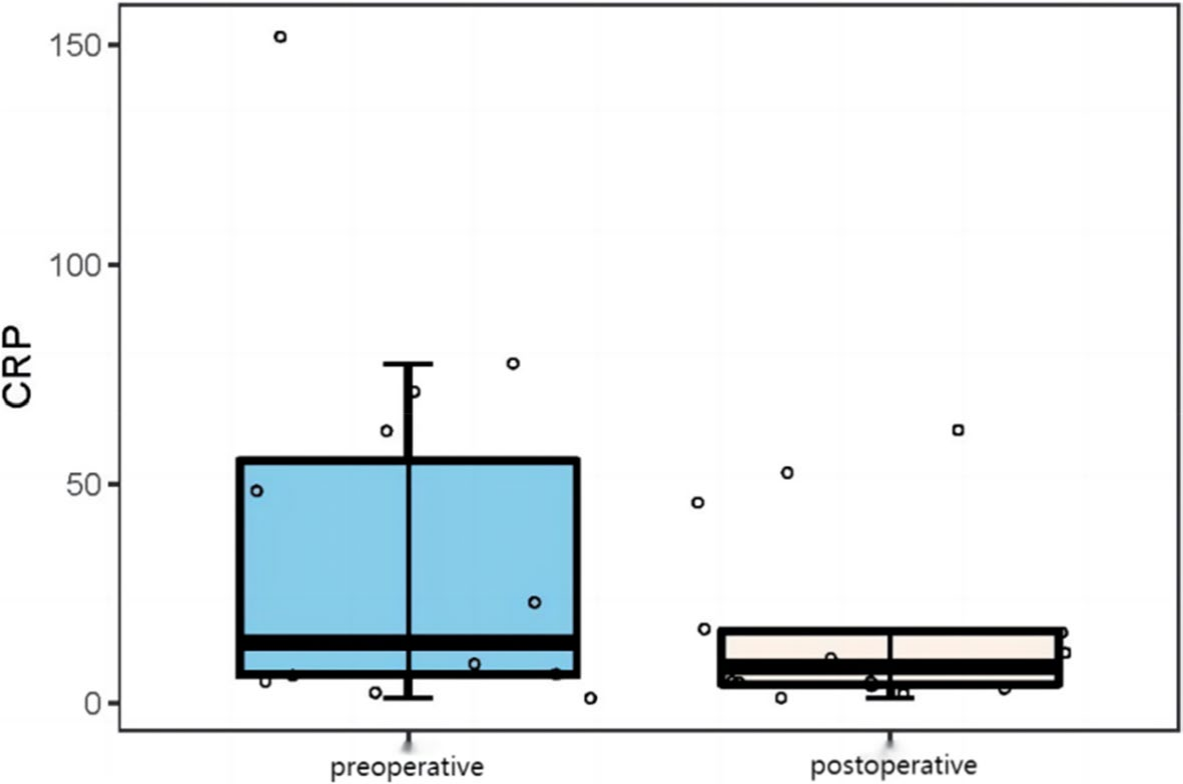
Analysis of CRP levels before and after surgery

**Fig. 5:**
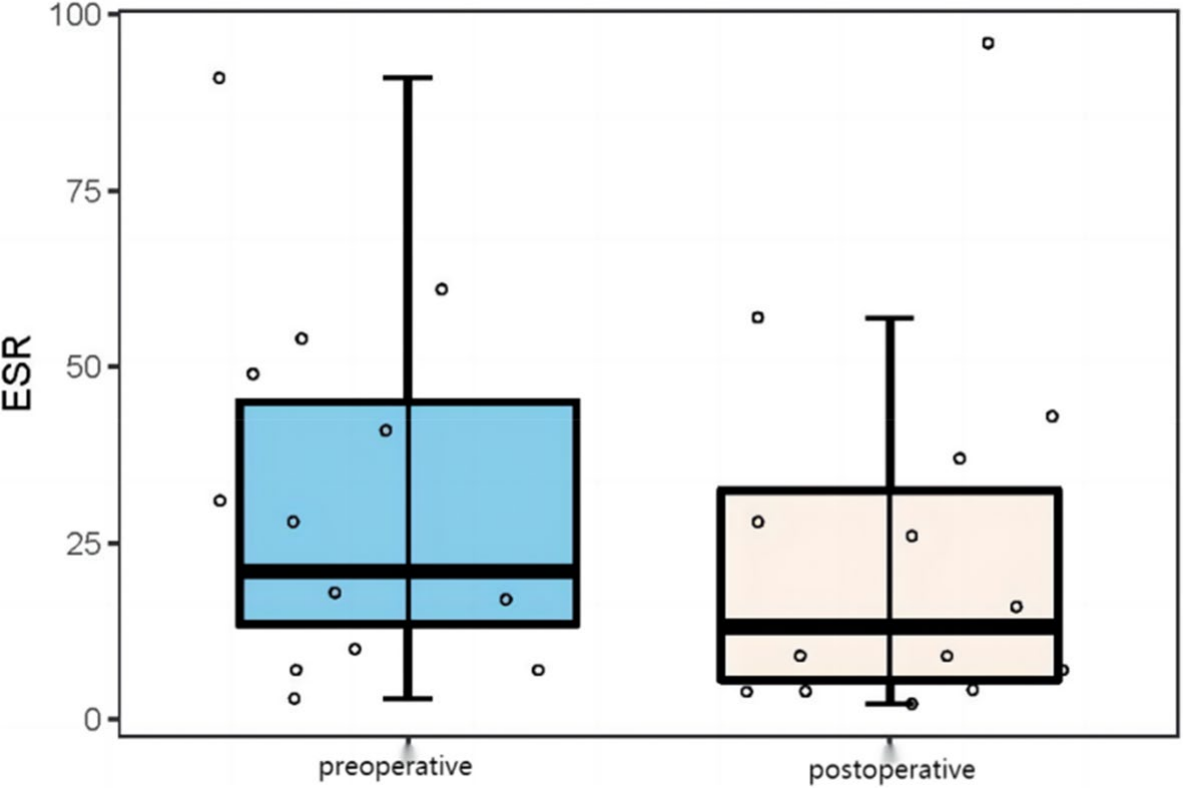
Summary of ESR levels before and after surgery

The results showed that the preoperative leukocyte count level (8.44 ± 2.71 × 10^9^/L vs 7.52 ± 3.49 × 10^9^/L), C-reactive protein (33.80 ± 41.86 mg/L vs 16.68 ± 19.92 mg/L) and sedimentation (30.60 ± 24.50 mg/L vs 23.69 ± 25.93 mg/L) were higher than the postoperative. The differences were all statistically significant (*P* < 0.05). The specific results are shown in (Table 3, Figs. 3, 4 and 5).

**Table 3:** Preoperative and postoperative analysis of serum inflammatory indicators

Serological indicators	Preoperative	Postoperative	*t*/*Z*	*P value*
WBC (10^9^ / L)	8.44 ± 2.71	7.52 ± 3.49	2.22	< 0.05
CRP (mg/L)	33.80 ± 41.86	16.68 ± 19.92	1.99	< 0.05
ESR (mg/L)	30.60 ± 24.50	23.69 ± 25.93	2.13	< 0.05

Serum inflammation index: white blood cell count (*WBC*), erythrocyte sedimentation rate (*ESR*), C-reactive protein (*CRP*) level

## Discussion

HO is a more common infectious disease of the bone and joint in children, with a higher incidence in developing countries compared to developed countries [9]. The clinical presentation is mostly characterised by redness, swelling and pain at the affected area, skin breakdown, sinus tract drainage and limited functional movement of the affected limb; a few cases may present with fever and vague pain, which is closely related to the degree of infection in the patient [10]. Early diagnosis and timely and effective treatment have a high cure rate, otherwise serious consequences will occur. For patients with HO, sensitive antibiotics are chosen according to the condition and for patients with bone defects, radical debridement combined with drug-laden calcium sulphate implantation is a good option.

In previous studies, it is considered that the surgical treatment of hematogenous osteomyelitis in children should be divided into 2 stages: (1) debridement, combined application of antibiotic-containing bone cement to osteomyelitis lesions, and temporary external fixation; and (2) Reconstruction of the bone defect. If the child has a concomitant pathological fracture, the fracture must be treated on the basis of antibiotics combined with debridement [1, 11].

Antibiotics combined with bone cement (bio-materials) for orthopedic treatment started in 1970, in a study by Buchholz [12] et al. Erythromycin, gentamicin and penicillin were first mixed with bone cement for fixing prostheses, and the implanted beads filled the cavity and controlled the local release of antibiotics forming a slow release system of antibiotics that was very effective in preventing infection around the prosthesis. With the development of basic science, different antibiotic carrier bio-materials have been generated. Currently, there are two types of antibiotic sustained release systems in clinical use: non-biodegradable sustained release systems and biodegradable sustained release systems.

Non-biodegradable, slow-release systems such as polymethylmethacrylate (PMMA) are the most common materials used to treat bone and joint conditions. The combination of antibiotics and PMMA for the treatment of osteomyelitis not only provides effective local control of bone infection, but also fills bone cavities and defects after debridement. It not only is anti-inflammatory but also has the effect of repairing bone defects to a certain extent, which can significantly improve the cure rate of osteomyelitis and shorten the course of the disease. However, PMMA also has certain limitations, first of all, PMMA is non-degradable and needs to be removed after implantation, which has the possibility of secondary damage and inducing secondary infection. In addition, The duration of the slow-release effect is a drawback, and some related studies have shown that the slow-release effect of PMMA antibiotics reaches its peak anti-infective function at 48–72 h after surgery, but a sharp decrease in local antibiotic concentration occurs after 72 h, and the PMMA carrier itself may induce bacterial colonization at antibacterial concentration or below, and the low concentration of antibiotics may promote bacterial resistance, leading to the recurrence of infection [13].

The shortcomings of PMMA have led to clinical research on biodegradable slow release systems. It has been found that CS has the characteristics of degradability, good biocompatibility, gradual and stable releasing of loaded antibiotics, and higher antibiotic elution rate compared with PMMA, without secondary surgery. In addition, calcium sulfate is similar in composition to human autologous bone, and the local space created by its degradation in vivo does not affect the migratory colonization of autologous osteoblasts, which has some osteogenesis-inducing effect, and calcium sulfate does not dissipate heat in vivo, which provides a wider choice of antibiotics [14].

PMMA and antibiotics mixing process due to the chemical reaction is easy to produce thermal effects, so the antibiotics used in combination with PMMA need to have good thermal stability. In addition, some antibiotics such as rifampin will destroy the polymerization reaction of PMMA, so PMMA can be loaded with a limited variety of antibiotics. Compared to PMMA, CS as an alternative biomaterial to PMMA is structurally stable, biocompatible, non-thermally effective and can encapsulate a wider range of antibiotics.CS can encapsulate water-soluble antibiotics such as glycopeptides and aminoglycosides (vancomycin) and fluoroquinolones (moxifloxacin or daptomycin, etc.). It has been shown that the use of CS loaded with tobramycin and vancomycin is effective in preventing the colonization and biofilm formation of MRSA and Staphylococcus epidermidis bacteria, and the use of CS loaded with broad-spectrum antibiotics has the function against Acinetobacter baumannii, Pseudomonas aeruginosa and Klebsiella pneumonia and can inhibit the biofilm formation of the corresponding pathogenic bacteria [15]. Zhao et al., reported 12 cases of acute hematogenous osteomyelitis caused by methicillin sensitive staphylococcus aureus (MSSA). In the treatment, The empirical use of antibiotics based on early diagnosis must first take the lead,and then reasonably select a sufficient number of sensitive antibiotics according to the results of drug sensitivity. Among them, oxacillin plays a crucial role in 3 severe patients with sepsis and lung abscess [16]. In a study by Melissa Depypere et al., the recommended use of vancomycin for MRSA infections, glycopeptides (vancomycin and teplane) and daptomycin both have low cure rates as experimental implant-associated infections however, daptomycin can be considered for cases of vancomycin allergy [17]. In this study, 15 patients with bacterial culture were MRSA infection, local use of drug-loaded calcium sulfate loaded vancomycin, which can not only achieve the effect of local anti-infection, but also promote the production of new bone, and can effectively control systemic infection, eliminate sepsis, whose effect is satisfactory.

CS, as an absorbable material, offers many advantages. First, it is stable and gradually absorbed, requiring no additional surgery for removal and avoiding secondary damage and infection; Second, antibiotic-impregnated CS has been found to be osteoinductive, inducing differentiation of bone marrow mesenchymal stem cells into osteoblasts and the formation of vascularised membranes, which not only prevents graft resorption but also promotes bone formation. Third, antibiotic-impregnated CS has been found to degrade at a rate comparable to that of newly generated bone; Fourth, CS is condidered as excellent antibiotic release kinetics. With the high compressive strength compared with PMMA and collagen sponge, fibrin and other new materials, CS has organic composition role and antibiotic release rate with safety. In contrast, the antibiotic release rate of collagen sponge is greater than its own decomposition rate, and too rapid release of antibiotics is likely to cause toxic side effects to the organism; fibrin antibiotic release rate is slightly slower than collagen sponge but the release time is shorter, and this class of biopolymers may be more suitable for acute infection control as well as chronic infection prevention, or as coating auxiliary materials to regulate the release rate of antibiotics from other materials [6, 7, 13, 15, 18–20].

The history of CS useed in pediatric hematogenous osteomyelitis is not long, but numerous clinical trials have found it to have many advantages and significant effects. As it has been widely used, there are still some limitations. The first is that it is less structurally stable than PMMA due to its degradability and does not provide stable support for larger bone defects after debridement, which is necessary for the healing of osteomyelitis. The second is the presence of sterile exudate, for instance, a common observation found in some studies of antibiotic-impregnated CS combined with herbal medicine for related treatment is the production of large amounts of water during calcium sulfate degradation resulting in sterile exudate from the wound or surgical site, occurring in 4%—51% of patients. How to prevent sterile exudation after the use of antibiotic-impregnated CS is an issue that needs to be addressed in the clinical use of antibiotic-impregnated CS for the treatment of hematogenous osteomyelitis. Most of the aseptic exudation was due to the exudation of calcium ions in CS after contact with soft tissues. Therefore, the author preserved the bone cortex covered with periosteum while thoroughly debriding, and filled the CS in a relatively airtight bone cavity to reduce the contact between CS and soft tissues, which could effectively reduce the aseptic exudation after surgery; Third, there is a possibility of complications. The majority of children in the TAO et al. study had a good prognosis, with one case of overgrowth and one case of overgrowth in the postoperative period [14]; Fourthly, higher brittleness and fragility; while the curing time is short and prepared in advance to reduce the time of implantation; The fifth is that the ideal antibiotic for impregnating CS is controversial. Any water-soluble antibiotic can be included in CS, with vancomycin, gentamicin and tobramycin being the usual choices for CS loading. In in vitro experiments, vancomycin and tobramycin-impregnated materials exhibited similar bactericidal properties and elution capacity. In one study, it was shown that the choice of tobramycin-containing CS or vancomycin combined with gentamicin-containing CS did not affect the eradication rate or the incidence of postoperative complications in patients with chronic osteomyelitis [8]. Based on previous experience, vancomycin is clinically selected for Gram-positive patients or patients with high suspicion of S. aureus infection, and gentamicin for Gram-negative patients; vancomycin and gentamicin are used together for patients with difficult-to-identify bacterial infections, but the choice of antibiotic still depends on the availability of accurate bacterial data, epidemiological data from the patient’s location, and progress in basic clinical research.

Antibiotic-impregnated CS has now been reported to be widely used in patients with post-traumatic osteomyelitis, post-operative infections resulting in osteomyelitis, and infections that do not heal in HO. Shi et al.’s performed a retrospective analysis systematically evaluating the eradication rates and associated postoperative complications of 16 clinical treatments for chronic osteomyelitis with drug-laden calcium sulphate. Fourteen of these studies were retrospective and 2 were prospective, enrolling 917 patients with an overall eradication rate of 92%. The treatment protocols in 15 of these studies all included radical debridement followed by implantation of drug-laden calcium sulphate and then adjunctive systemic antibiotics, with a good prognosis [13]. Andreacchio A et al. reported on the treatment of 12 patients with chronic osteomyelitis with tobramycin-impregnated calcium sulphate in combination with topical administration of 4% tobramycin calcium sulphate granules and intravenous antibiotics after thorough debridement, with satisfactory results and reduced co-morbidity, with eradication of infection and bone healing observed during follow-up [21]. Ellur et al. retrospectively evaluated a consecutive series of 34 patients studied from 2011 to 2017 in which the defect cavity created in the bone was filled with antibiotic-impregnated calcium sulphate prior to initial closure following thorough surgical debridement. Of the 31 patients available for follow-up, effective bone regeneration was confirmed in all cases and radiological bone healing was usually observed at around 12 weeks. At the final follow-up, all patients were free of infection and reoperation. CS was completely resorbed within 3 months. No systemic adverse reactions to locally delivered antibiotics were observed [22]. Tao et al. conducted an evaluation of the efficacy of antibiotic-impregnated calcium sulfate for the treatment of pediatric hematogenous osteomyelitis based on previous work, and cumulatively included 21 patients with pediatric hematogenous osteomyelitis treated at this institution from 2013–2018 for evaluation. Observing and analyzing relevant indicators, they found a 0% (0/21) recurrence rate of infection after a long-term follow-up (31–91 months), They also found that the infection was eradicated in all 21 patients treated with antibiotic-impregnated CS and none required reoperation, confirming the therapeutic efficacy of antibiotic-impregnated CS in eradicating the infection [14]. Numerous studies have amply demonstrated the superior therapeutic efficacy of antibiotic-impregnated CS in paediatric haematogenous osteomyelitis.

All pediatric patients in this study were surgically implanted with drug-laden calcium sulfate, and the preoperative serum inflammatory indexes were statistically analyzed to be higher than the postoperative ones, and all differences were statistically significant. The vulnerable site of hematogenous osteomyelitis in children is the long bone epiphysis, once diagnosed a thorough debridement of the infected lesion should be performed promptly to reduce bacterial colonization of the infected site, Debridement also reduces the risk of local inflammatory reaction and the occurrence of chronic osteomyelitis; a thorough debridement will leave bone defect [17] and there will be some stimulation of the epiphysis.

In all children with hematogenous osteomyelitis, the infected lesions are cleared. For bone defects after debridement, drug-loaded calcium sulfate is a good choice. Medical calcium sulphate (calcium sulphate) both eliminates bone defects and dead spaces and carries antibiotics to treat the infection, while CS itself is osteogenic and repairs the bone defects in one stage. All patients in this study recovered well after surgery, without the need for reoperation, and without infection or complications. The efficacy of the drug-loaded calcium sulfate in the treatment of hematogenous osteomyelitis in children was confirmed.

There is no doubt that CS is promising in pediatric hematogenous osteomyelitis, while there are shortcomings to be refined in the subsequent use and research. First, implanting CS in a wet environment should be avoided because CS does not solidify well after mixing and can easily dissolve when it meets blood, leading to the failure of treatment means. Therefore, it is important to stop bleeding thoroughly before implantation, keep the implant space dry, and prepare the CS in strict accordance with the instructions to prevent the CS from becoming too wet or completely cured for implantation to become unshapable. Placement of the CS within a relatively closed bone structure at the time of surgery, or if this is not possible, an area rich in soft tissue can be implanted and postoperative drainage can be placed for 1–2 weeks; Secondly, always pay attention to the clinical dynamics of the patient to adjust the whole treatment plan to reduce the possibility of complications; The third is to improve the precision of diagnosis and treatment by obtaining accurate bacterial data, epidemiological data of the patient’s location, and keeping up with advances in basic clinical research to select the best antibiotic type and concentration for each situation; Fourth, we should devote to exploration of composite materials. As a single calcium sulphate system for the release of antibiotics and a new system for the delivery of materials both have certain shortcomings, we can try to combine natural or synthetic polymers with CS to form a material with both toughness, osteoinductivity and sustained antibiotic release properties in subsequent studies [18]. The high compressive strength of CS can play a role in structural support, and the polymer composite can improve its toughness, as well as modulate the rate of antibiotic release. In addition, polymers can also enhance the intercellular interactions between trauma and between material and trauma, effectively improving their biological properties.

Our study also has some limitations. Firstly this study was not a retrospective controlled study and there was no control group to compare. Secondly, the study was a small sample size, only 15 patients were included. Finally the site of bone defects involved different skeletal sites.

## Data Availability

The results of this study were obtained from the Fourth Affiliated Hospital of Xinjiang Medical University.The data analyzed during the present study are all available from the corresponding author on reasonable request.

## References

[1] Woods CR, Bradley JS, Chatterjee A (2021). Clinical practice guideline by the Pediatric Infectious Diseases Society and the Infectious Diseases Society of America: 2021 guideline on diagnosis and management of acute hematogenous osteomyelitis in pediatrics. J Pediatric Infect Dis Soc.

[2] Choi IH, Cho TJ, Moon HJ (2011). Ilizarov treatment of congenital pseudarthrosis of the tibia: a multi-targeted approach using the Ilizarov technique. Clin Orthop Surg.

[3] Zairi M, Mohseni AA, Msakni A (2022). Acute hematogenous osteomyelitis in children: Management of pandiaphysitis with extensive bone destruction: A case series of thirteen child. Ann Med Surg.

[4] Riise ØR, Kirkhus E, Handeland KS (2008). Childhood osteomyelitis-incidence and differentiation from other acute onset musculoskeletal features in a population-based study. BMC Pediatrics.

[5] Harik NS, Smeltzer MS (2010). Management of acute hematogenous osteomyelitis in children. Expert Rev Anti Infect Ther.

[6] McConoughey SJ, Howlin RP, Wiseman J (2015). Comparing PMMA and calcium sulfate as carriers for the local delivery of antibiotics to infected surgical sites. J Biomed Mater Res - Part B Appl Biomater.

[7] Oh EJ, Oh SH, Lee IS (2016). Antibiotic-eluting hydrophilized PMMA bone cement with prolonged bactericidal effect for the treatment of osteomyelitis. J Biomater Appl.

[8] McKee MD, Li-Bland EA, Wild LM (2010). A Prospective, Randomized Clinical Trial Comparing an Antibiotic-Impregnated Bioabsorbable Bone Substitute With Standard Antibiotic-Impregnated Cement Beads in the Treatment of Chronic Osteomyelitis and Infected Nonunion. JorthopTrauma.

[9] Dartnell J, Ramachandran M, Katchburian M. Haematogenous acute and subacute paediatric osteomyelitis: a system aticreview of the literature. J Bone Joint Surg Br. 2012;94(5):584–95. 10.1302/0301-620X.94B5.28523 22529075

[10] Dodwell ER. Osteomyelitis and septic arthritis in children: current concepts. Curr Opin Pediatr. 2013;25(1):58–63. 10.1097/MOP.0b013e32835c2b42 23283291

[11] Yildirim A, Kapukaya A, Atiç R (2017). The Use of an "internal Fixator Technique" to Stabilize Pathologic Fractures Developing Secondary to Osteomyelitis. J Pediatr Orthop.

[12] Baumbach SF, Hobohm L, Wozasek GE (2011). A treatment strategy for complex cases of osteomyelitis in children and its applicability on three exemplary cases. J Pediatr Orthop Part B.

[13] Shi X, Wu Y, Ni H (2022). Antibiotic-loaded calcium sulfate in clinical treatment of chronic osteomyelitis: a systematic review and Meta-analysis. J Orthop Surg Res, BMC.

[14] Tao R, Wu JQ, Luo JW (2022). Antibiotic-impregnated calcium sulfate for the treatment of pediatric hematogenous osteomyelitis. BMC Pediatr.

[15] Luo S, Jiang T, Yang Y (2016). Combination therapy with vancomycin-loaded calcium sulfate and vancomycin-loaded PMMA in the treatment of chronic osteomyelitis. BMC Musculoskelet Disord.

[16] Zihou Z, Guoliang W, Yanjun W (2020). Analysis of systemic and local antibiotic treatment for acute hematogenous osteomyelitis [J]. Adv Modern Biomed.

[17] Depypere Melissa, Kuehl Richard, Metsemakers Willem-Jan (2020). Recommendations for Systemic Antimicrobial Therapy in Fracture-Related Infection: A Consensus From an International Expert Group. J Orthop Trauma.

[18] Qin CH, Zhang HA, Chee YH (2019). Comparison of the use of antibiotic-loaded calcium sulphate and wound irrigation-suction in the treatment of lower limb chronic osteomyelitis. Injury.

[19] Wang B, Cheng W, Liu F (2022). Efficacy and safety of vancomycin-loaded calcium sulfate versus conventional surgical debridement for pediatric acute osteomyelitis: a retrospective study. BMC Musculoskelet Disord.

[20] Xiang H, Wang Y, et al. Cerium-containing a-calcium sulfate hemihydrate bone substitute promotes osteogenesis. J Biomater Appl. 2019;34(2):250–60. 10.1177/0885328219849712 31088183

[21] Andreacchio A, Alberghina F, Paonessa M (2018). Tobramycin-impregnated calcium sulfate pellets for the treatment of chronic osteomyelitis in children and adolescents.

[22] Ellur V, Kumar G, Sampath JS (2021). Treatment of chronic hematogenous osteomyelitis in children with antibiotic impregnated calcium sulphate. J Pediatr Orthoped.

